# The informatics pathway for hospital infection quality control monitoring

**DOI:** 10.3389/fpubh.2025.1543375

**Published:** 2025-03-05

**Authors:** Hui Wang, Qin Huang, Qingqing Tian, Weiwei Yang, Anran Liu, Jiayang Tang, Hailin Zhang, Chunlin Wu

**Affiliations:** Department of Hospital Infection Control and Public Health, Sichuan Cancer Hospital, Chengdu, China

**Keywords:** hospital infection monitoring, information system, quality control, missed reports, report card quality

## Abstract

**Objective:**

To analyze the results and current status of hospital infection monitoring and quality control work at a tertiary specialized cancer hospital, providing references for further improvement of HAI monitoring quality.

**Methods:**

Combined information-based monitoring with manual quality control measures for hospital infection surveillance, collecting data such as hospital infection report cards and missed reports to analyze the current status of hospital infection monitoring. Starting from January 2023, further comprehensive quality control measures were taken, and data before and after the implementation were compared to evaluate the effectiveness of the quality control work.

**Results:**

After the implementation of comprehensive management measures, the incidence rate of hospital infection in the hospital decreased from 0.60 to 0.52%, the correct rate of early warning disposal increased from 85.31 to 89.53%, and the handling rate within 24 h of early warning increased from 20.51 to 72.99%. All these differences are statistically significant (*p* < 0.05). After the comprehensive management measures were adopted, more missed cases were detected and reported, resulting in an increase in the missed reporting rate from 5.36 to 12.33% (*p* < 0.05). While the number of reports increased, the accuracy of the reports decreased from 67.54 to 54.05% (*p* < 0.05).

**Conclusion:**

Information systems can enhance the efficiency of hospital infection surveillance through real-time monitoring and automatic early warning, improve the quality of reporting, and thereby contribute to the reduction of the incidence rate of hospital infections. However, the quality of monitoring is still influenced by human factors such as whether the early warning rules are scientifically set and whether the determination of hospital infections is accurate. There may be situations where the missed reporting rate is underestimated and the quality of the report cards is not high. This indicates that while adopting information-based monitoring, we cannot ignore the management of quality control. It is necessary to continuously strengthen the investigation of missed reporting, improve the quality of report cards, and ensure the authenticity and accuracy of the results of hospital infection monitoring.

## 1 Introduction

Hospital infections, also known as hospital-acquired infections (HAIs), refer to infections that patients acquire within a hospital setting, including those that occur during hospitalization as well as those that manifest after discharge but were acquired during the hospital stay. HAIs not only increase the economic burden on patients but also pose significant threats to their health and lives (1). Surveillance of HAIs is a crucial measure for their prevention, control, and management (2), enabling healthcare facilities to promptly identify issues, implement interventions, and assess their effectiveness, thereby effectively preventing the occurrence of HAIs (3). Traditionally, the monitoring of HAI cases often involved manually reviewing paper medical records, a method that is inefficient and prone to delays (4–6). The good news is that in recent years, medical professionals have explored an Informatics Pathway for hospital infection surveillance: that is, through the Hospital Infection Surveillance Information System, relevant patient infection data is collected in real-time, infection risks are automatically assessed, and early warnings are generated to assist medical staff in quickly identifying hospital infection cases. The widespread adoption of various HAI surveillance information systems has enhanced early warning capabilities and significantly improved the level of HAI monitoring (7, 8). Studies have shown that HAI surveillance systems help reduce the incidence and underreporting rates of HAIs (3, 9–11), although they cannot completely eliminate underreporting. Additionally, the quality of HAI case report forms is a critical factor influencing the effectiveness of surveillance, often relying heavily on manual quality control measures. Currently, there is a paucity of research in China on the quality of HAI case report forms. In summary, while information systems facilitate surveillance through early infection warnings, their accuracy is influenced by the precision of alerts, the appropriateness of physicians’ responses to these alerts, and the quality of the case report forms. These systemic and human factors can lead to the underestimation of HAI underreporting rates, delays in alert management, and inaccuracies in case reporting, all of which impact the reliability of HAI surveillance data. Therefore, this study statistically analyzes the HAI surveillance data of a tertiary cancer specialty hospital, examining HAI incidence rates, underreporting rates, and results related to the quality control of case report forms. The aim is to gain a deeper understanding of the monitoring effectiveness of the HAI information system, explore the impact of comprehensive quality control measures on surveillance outcomes, and accurately assess the current status of HAIs, providing a reference for the high-quality conduct of future monitoring efforts.

## 2 Methods

### 2.1 Data sources

The hospital infection surveillance and quality control management data were collected from inpatients at our hospital between January 2022 and December 2023.

### 2.2 Research methods

#### 2.2.1 Establishment of a hospital infection surveillance information system

A hospital infection monitoring system developed by a designated company was employed. This system integrates with various hospital subsystems, such as the Hospital Information System (HIS), imaging systems, and Laboratory Information System (LIS), to automatically collect real-time information related to inpatients. The monitoring system has established a series of alert-triggering conditions, which are mainly abnormal events related to infections in patients, such as fever, the use of antimicrobial agents, positive culture results from specimens, and abnormal white blood cell counts, among others. If the system detects these abnormal events in a patient, an infection alert will be generated. The more abnormal events detected, the higher the suspicion level of the infection alert for that patient. These alerts are categorized into four levels of suspicion: 30, 50, 70, and 90%. The higher the suspicion level, the greater the likelihood of hospital-acquired infection in the patient.

#### 2.2.2 Processing of infection alerts

The hospital requires doctors to review and handle infection alerts daily and to track the timeliness rate of alert processing. Hospital Infection Prevention and Control Dedicated Staff pre-process all 90% suspicion-level alerts on a weekly basis. In accordance with the “Diagnostic Criteria for Hospital Infections “issued by the Ministry of Health of China, they determine whether to confirm or rule out hospital infections and document the results of the pre-processing. Attending physicians are also required to promptly handle 90% suspicion-level alerts in their respective departments and compare their results with those of the designated personnel. If there is any discrepancy between the conclusions reached by the designated personnel and the attending physicians, a discussion is conducted, and the mutually agreed-upon result is taken as the final conclusion. For alerts with suspicion levels below 90%, attending physicians independently determine whether to diagnose or rule out hospital infections based on the patient’s clinical status.

#### 2.2.3 Classification of alert handling outcomes

Errors in alert handling by physicians are categorized into four types: underreporting, diagnostic errors, insufficient evidence, and external infections, defined as follows: (1) Underreporting: when a patient has a hospital-acquired infection but the attending physician erroneously excludes it. (2) Diagnostic errors: the patient has a hospital-acquired infection, and although the physician correctly determines that the patient has a hospital infection, the site/type of infection is incorrectly reported on the hospital infection report card. (3) Insufficient evidence: when the attending physician diagnoses a hospital-acquired infection despite insufficient evidence supporting the diagnosis. (4) External infection: when the attending physician diagnoses a hospital-acquired infection, but the infection is actually acquired outside the hospital.

#### 2.2.4 Quality control of hospital infection report forms

Designated personnel periodically review the hospital infection report forms submitted by physicians according to hospital infection diagnostic standards, recording the audit results. The categories of audit results—external infections, insufficient evidence, and diagnostic errors—are consistent with the definitions in section 2.2.3.

### 2.3 Comprehensive quality control measures

Starting from January 2023, comprehensive quality control measures were implemented for hospital infection surveillance management. These measures include: (1) Monthly hospital infection quality control meetings to report and address surveillance-related issues; (2) Incorporating underreporting of hospital infections into defect management, including point deductions and fines; (3) Including hospital infection surveillance as part of the hospital infection quality control assessment, linking it to departmental performance evaluations; (4) Intensifying supervision by conducting on-site discussions or communicating via WeChat or phone with departments and physicians who have higher numbers of issues related to hospital infection surveillance.

In addition, some intervention measures have also been adopted, such as expanding the scope of alert sampling to include 30, 50, and 70% suspicion levels for infection alerts that were ruled out by physicians, and recording the quality control outcomes.

### 2.4 Evaluation metrics

Hospital Infection Underreporting Rate = (Number of underreported HAI cases identified / Total number of HAI cases that should have been reported) × 100%.

Correct Alert Handling Rate = (Number of infection alerts correctly handled by attending physicians / Total number of infection alerts sampled) × 100%.

Correct Report Form Rate = (Number of correctly reported HAI forms / Total number of HAI forms submitted) × 100%.

### 2.5 Statistical analysis

Data entry and organization were performed using Excel 2010. Statistical analyses were conducted using SPSS version 21.0. Chi-square tests were employed for the comparison of various rates, with the significance level (*α*) set at 0.05.

### 2.6 Ethical approval and informed consent

This study has passed the ethical review by the Sichuan Cancer Hospital Ethics Committee and adheres to the principles of the Declaration of Helsinki.

This study utilizes data extracted from the hospital information system related to hospital infection monitoring to assess and enhance the effectiveness of infection monitoring practices. All patient data used in this study was rigorously anonymized during extraction, with no personal privacy information involved. The study does not entail any direct intervention with patients or the collection of personal privacy data.

According to the review and approval by the Ethics Committee of Sichuan Cancer Hospital (Approval No: SCCHEC-02-2024-033), this study meets the criteria for an ethical waiver, although the study involves the use of data related to human subjects, it does not involve the direct collection of personal privacy information, any form of patient intervention, or any commercial interest. Consequently, additional informed consent from each patient is not required.

### 2.7 Approval of experimental protocols

The Sichuan Cancer Hospital Ethics Committee has approved the experimental protocols. All methods used in this study were carried out in accordance with relevant guidelines and regulations.

## 3 Results

### 3.1 Incidence of hospital-acquired infections (HAIs)

In 2022, the total number of inpatients was 69,897, with 421 cases of HAIs, resulting in an incidence rate of 0.60%. In 2023, the number of inpatients increased to 81,009, with 418 HAI cases, leading to an incidence rate of 0.52%. A comparison of the incidence rates between the 2 years yielded a chi-square value of 5.057, with a *p*-value of 0.025, indicating a statistically significant difference.

### 3.2 Handling of infection alerts

From 2022 to 2023, designated personnel investigated a total of 1,642 infection alerts. Among these, 387 cases were confirmed as HAIs, accounting for 23.57% of the total alerts. Specifically, 1,365 alerts with a suspicion level of 90% were investigated, and 384 cases of HAIs were identified, representing 28.13% of these high-suspicion alerts. Additionally, 277 alerts with suspicion levels below 90% were randomly sampled, yielding 3 HAI cases, accounting for 1.08%. In 2022, the correct handling rate of alerts was 85.31%, and in 2023, it was 89.53%, with a chi-square value of 6.126 and a *p*-value of 0.013. The difference is statistically significant, indicating an increase in the correct handling rate of alerts in 2023. Details are provided in Table 1.

**Table 1:** Analysis of infection alert handling outcomes.

Year	Number of alerts sampled	Number of HAI cases	Percentage of HAI cases (%)	Underreporting	External infections	Diagnostic errors	Insufficient evidence	Correctly handled cases	Correct handling rate (%)
2022	524	168	32.06	9	14	33	21	447	85.31
2023	1,118	219	19.59	27	37	29	24	1,001	89.53
Total	1,642	387	23.57	36	51	62	45	1,448	88.19

### 3.3 Underreporting investigation results

From 2022 to 2023, a total of 387 HAI cases were identified through random sampling by designated personnel, among which 36 cases were underreported by physicians, resulting in an underreporting rate of 9.30%. A comparison of the underreporting rates between 2022 and 2023 yielded a chi-square value of 5.477 and a *p*-value of 0.019, indicating a statistically significant difference. This suggests that the HAI underreporting rate increased in 2023 compared to 2022. Details are provided in Table 2.

**Table 2:** Results of underreporting investigation.

Year	Underreported cases	Non-Underreported cases	Total cases	Underreporting rate (%)
2022	9	159	168	5.36
2023	27	192	219	12.33
Total	36	351	387	9.30

### 3.4 Analysis of timeliness in alert handling

A comparison of the 24-h and 48-h alert handling rates between 2022 and 2023 showed chi-square values of 5091.01 (*p* < 0.001) and 4017.25 (*p* < 0.001), respectively, indicating statistically significant differences. These results suggest that the implementation of enhanced quality control measures improved the timeliness of alert handling. Details are provided in Table 3.

**Table 3:** Analysis of alert handling and timeliness.

Year	Number of alerts	Alerts handled Within 24 hours	24 h handling rate (%)	Alerts handled within 48 hours	48 h handling rate (%)
2022	6,191	1,270	20.51	1819	29.38
2023	16,304	11,900	72.99	12,256	75.17

### 3.5 Quality control analysis of hospital infection report forms

Over the 2 years, a total of 1,239 hospital infection report forms were submitted, of which 492 were identified as problematic, resulting in a correct reporting rate of 60.29%. Details are provided in Table 4. A comparison of the correct reporting rates between 2022 and 2023 yielded a chi-square value of 16.943 (*p* < 0.001), indicating a statistically significant difference. The correct reporting rate declined in 2023.

**Table 4:** Quality control analysis of hospital infection report forms.

Year	External infections	Insufficient evidence	Diagnostic errors	Total problematic forms	Total submitted forms	Correct reporting rate (%)
2022	58	97	31	186	573	67.54
2023	128	107	71	306	666	54.05
Total	186	204	102	492	1,239	60.29

Among the 102 report cards with diagnostic errors for infections, the type of hospital-acquired infection most frequently misdiagnosed was lower respiratory tract infection (47 cases, 46.08%), which doctors often incorrectly reported as ventilator-associated pneumonia or upper respiratory tract infection; followed by bloodstream infections (17 cases, 16.67%), which were often mistakenly diagnosed as catheter-related infections or intra-abdominal tissue infections; additionally, surgical site infections (14 cases, 13.73%) were commonly misdiagnosed as skin and soft tissue infections. Details are provided in Table 5.

**Table 5:** Analysis of common diagnostic errors in hospital-acquired infections.

Actual diagnosis	Physician’s reported diagnosis	Number of cases	Total misdiagnosed cases
Lower respiratory tract infection	Upper respiratory infection (URI)	24	47
	Ventilator-associated pneumonia (VAP)	9	
	Abdominal tissue infection	6	
	Others	8	
Bloodstream infection	Central line-associated bloodstream infection (CLABSI)	5	17
	Abdominal tissue infection	4	
	Urinary system infection	3	
	Gastrointestinal infection	3	
	Others	2	
Surgical site infection	Soft tissue infection	5	14
	Skin infection	3	
	Others	6	

## 4 Discussion

The incidence of hospital-acquired infections (HAIs) varies across different regions, types, and levels of healthcare institutions (5, 9, 12). In this study, the implementation of comprehensive quality control measures led to a reduction in the HAI incidence rate. The author analyzed that the implementation of quality management measures has further raised the awareness of hospital staff regarding the importance of hospital infection control, enhanced their willingness and capability to implement infection control measures, and thereby reduced the incidence rate of hospital infections. Case alert functionality is a fundamental component of infection surveillance systems, enabling the identification of potential HAI cases to minimize underreporting (13). In this study, 1,365 infection alerts with a 90% suspicion level were investigated by dedicated personnel, among which 384 cases were confirmed as HAIs, yielding a positive predictive value (PPV) of 28.13%, which is less than 30%. This differs from similar studies (14–16), suggesting the need to further improve the PPV of HAI alerts to reduce the workload of medical staff. Additionally, among the 277 infection alerts with suspicion levels below 90% that were sampled, 3 HAI cases were identified, accounting for 1.08%. Although the proportion is low, given the large volume of alerts with suspicion levels below 90%, the number of underreported cases could be substantial if these alerts are not investigated, leading to an underestimation of the true underreporting rate. Therefore, healthcare institutions must further optimize surveillance system logic and enhance the scientific rigor of HAI alert criteria.

Previous studies have reported varying results regarding the underreporting rates of HAIs (11, 14, 17), highlighting that underreporting remains common in surveillance systems based on digital information, and accurately assessing it poses certain challenges. In this study, the comparison of underreporting rates between 2022 and 2023 revealed a statistically significant difference (*p* = 0.019), indicating an increase in the underreporting rate in 2023 compared to 2022. The likely reason for this increase, as analyzed by the authors, is the expanded scope of sampling due to the implementation of comprehensive quality control measures in 2023, which led to the identification of more underreported cases, causing short-term fluctuations in the underreporting rate. This is not due to an increase in erroneous exclusions by physicians, as evidenced by the improvement in the correct handling rate in 2023. This finding suggests that the underreporting rate may have been underestimated before the implementation of comprehensive quality control measures. Further observation is needed to evaluate the long-term effects of these measures on reducing underreporting rates (18).

In addition to underreporting, the quality of hospital infection report forms also plays a vital role in the reliability of HAI surveillance data. High-quality report forms accurately reflect the actual status of HAIs and help infection control personnel identify critical departments, processes, and sites for targeted interventions. In this study, the correct reporting rate in 2023 showed a statistically significant decline compared to 2022. Currently, there is limited research, both domestically and internationally, on the impact of hospital infection management on the quality of HAI report forms. Based on general experience in infection control, comprehensive quality control measures should positively influence the quality of report forms; however, this study did not confirm this expectation. Possible reasons for this discrepancy include the following: (1) The intensified quality control measures penalized underreporting, increasing physicians’ awareness and frequency of report form submission, but without adequate emphasis on the quality of these forms, leading to a decline in accuracy. Additionally, more meticulous audits led to the identification of a greater number of problematic report forms. (2) The current HAI diagnostic criteria in China have not been updated for a long time, resulting in numerous cases where the criteria are not applicable in practice. Different healthcare providers may interpret the criteria differently, leading to inconsistencies in judgment. (3) Clinical physicians often have busy schedules and limited time, leading to inadequate focus on HAI-related training, which may not have achieved its intended effect.

This study has certain limitations. For example, infection alerts with a suspicion level below 90% were not fully investigated, instead, a sampling approach was used, and cases without alerts were not considered. This may have led to the underreporting of potential HAI cases, resulting in underestimations of both the HAI incidence and underreporting rates.

## 5 Conclusion

The findings of this study suggest that hospital infection surveillance information systems cannot completely eliminate the issue of underreporting, underscoring the need for continuous enhancement of quality control management. Comprehensive quality control measures can help reduce the incidence of hospital-acquired infections (HAIs), improve the accuracy and timeliness of alert handling, and help identify more underreported cases. Dedicated personnel should focus on the quality of HAI report forms and intensify the auditing process. Additionally, improving the quality of HAI surveillance requires continuously refining quality control assessment systems, emphasizing the effectiveness of training, and optimizing the alert functionalities of monitoring systems. The short-term impact of quality control measures on HAI surveillance indicators may show fluctuations, and evaluating their true effectiveness necessitates long-term observation and practice.

## Data Availability

The dataset used in this study has not been publicly released. However, we are willing to share the data with other researchers upon reasonable request. Researchers interested in accessing the dataset can contact us via email at wanghui1@scszlyy.org.cn, and we will consider the feasibility of providing the data based on the specifics of the request.
